# Effect of UVC Radiation on Hydrated and Desiccated Cultures of Slightly Halophilic and Non-Halophilic Methanogenic Archaea: Implications for Life on Mars

**DOI:** 10.3390/microorganisms6020043

**Published:** 2018-05-12

**Authors:** Navita Sinha, Timothy A. Kral

**Affiliations:** 1 Arkansas Center for Space and Planetary Sciences, University of Arkansas, Fayetteville, AR 72701, USA; navitasinha0919@gmail.com; 2 Department of Biological Sciences, SCEN-601, University of Arkansas, Fayetteville, AR 72701, USA

**Keywords:** methanogens, Mars, UVC radiation, halophiles

## Abstract

Methanogens have been considered models for life on Mars for many years. In order to survive any exposure at the surface of Mars, methanogens would have to endure Martian UVC radiation. In this research, we irradiated hydrated and desiccated cultures of slightly halophilic *Methanococcus maripaludis* and non-halophilic *Methanobacterium formicicum* for various time intervals with UVC (254 nm) radiation. The survivability of the methanogens was determined by measuring methane concentrations in the headspace gas samples of culture tubes after re-inoculation of the methanogens into their growth-supporting media following exposure to UVC radiation. Hydrated *M. maripaludis* survived 24 h of UVC exposure, while in a desiccated condition they endured for 16 h. *M. formicicum* also survived UVC radiation for 24 h in a liquid state; however, in a desiccated condition, the survivability of *M. formicicum* was only 12 h. Some of the components of the growth media could have served as shielding agents that protected cells from damage caused by exposure to ultraviolet radiation. Overall, these results suggest that limited exposure (12–24 h) to UVC radiation on the surface of Mars would not necessarily be a limiting factor for the survivability of *M. maripaludis* and *M. formicicum*.

## 1. Introduction

### Mars UV Radiation

Ultraviolet (UV) flux on a celestial body is one of the significant constraints that affect the evolution of life on that system. The current Martian UV flux is much stronger and harsher [1,2,3,4,5] than the present Earth UV flux. The reason is that Mars lacks a significant global ozone layer and a magnetic field. The ozone layer in the atmosphere absorbs harmful UV radiation of the Sun and prevents it from reaching the surface, and the magnetic field deflects energetic charged particles such as galactic cosmic rays (GCRs) and solar energetic particles (SEPs), and blocks particles from incident on the surface. In addition to this, the Martian atmosphere is much thinner than that of the Earth’s atmosphere contributing to the higher UV flux on the surface of Mars. The solar constant of Mars is only 43% of that found on Earth, mostly in the ultraviolet region of 200–300 nm [6]. Below ~190 nm, the radiation is attenuated by Martian atmospheric CO_2_. However, it has been suggested that Earth and Mars shared a similar geological history [7]. Before the rise of oxygen and ozone in the Earth atmosphere, life was probably exposed to higher doses of UV radiation than that of the present day Earth [8,9]. The DNA damaging UV irradiation on the surface of present day Mars is analogous to that of the Archaean Earth. Hence, UV flux may not be a critical limiting factor for the evolution of life on Mars [10], but the combination of several environmental factors such as low temperature, lack of liquid water, and the presence of oxidizing compounds cause the surface of Mars to be currently inhospitable.

In spite of the hostile surface conditions of present-day Mars, there is a possibility that life could have evolved on early Mars and may still be present today. Early Martian life could have left biosignatures on Mars as well. Furthermore, with the discovery and the study of several types of Earth extremophiles [11] and the occurrence of transient liquid water on Mars [12], it is not prudent to say that life is impossible on the surface of contemporary Mars. Interestingly, the discovery of methane in the Martian atmosphere [13,14,15,16] further bolstered the perception of the possibility of life on Mars. On Earth, 90–95% of atmospheric methane has a biological origin [17]. One of the sources of Earth’s atmospheric methane is methane-producing Archaea, known as methanogens. Methanogens have been considered ideal candidates for life on Mars for a long time. Our exobiology lab research group has been studying the survivability of methanogens under different conditions approaching those on Mars even before the discovery of methane in the Martian atmosphere [18,19,20].

The chemistry of Mars supports the possibility of life [21]. The presence of C, H, N, O, P, S, heavy metals, and minerals [22] provide essential ingredients for the building blocks of life, for example, for the synthesis of proteins and DNA. Additionally, the presence of various forms of salts such as sulfate, and perchlorates found on the surface of Mars [23,24,25] could not only support some forms of life, but could also decrease the freezing point and the evaporation rate of water. For these reasons, salt-loving microorganisms called halophiles are also considered as a model life form on Mars [26]. Halophiles are classified as extremophiles that can survive and thrive in high salt concentrations and low water activity environments. Even some haloarchaea have been isolated from rock salt samples that are hundreds to millions of years old [27,28], which suggests that halophiles can survive for a long time under desiccated salty environments. However, the mechanisms responsible for the extreme longevity of archaea in the dormant stage are not known because archaea do not form spores [29], although some do form cyst-like cells [30].

Ultraviolet (UV) radiation wavelengths range from 10 to 400 nm in an electromagnetic spectrum. The wavelengths below 400 nm are harmful, mutagenic or even lethal for most of Earth’s life. The short wavelengths of UV, such as UVB (280–315 nm) and UVC (200–280 nm), cause lesions in DNA molecules of a biological system resulting in temporary or permanent mutations. A covalent bond is formed between two adjacent thymine or cytosine molecules called pyrimidine dimers [31], and DNA polymerase cannot replicate beyond the site of dimer formation, resulting in cell death. On Mars, the UV flux reaching its surface is mostly between 190 and 400 nm. Therefore, life on Mars would have to adapt to UV radiation by using DNA repair mechanisms. Different types of DNA repair systems, such as photoreactivation, excision repair, and recombination repair [32,33,34], have been reported in Archaea. Moreover, the effect of Martian UV radiation on a biological system can be attenuated by the presence of clouds, dust, ice, water, and minerals. Several physical and chemical ultraviolet screening compounds have been reported including halite—solid NaCl [35,36].

A vast body of literature is available about the growth and survivability of different types of microorganisms under simulated Martian physical and chemical conditions including ultraviolet radiation [37,38,39]. Methanogens and halophiles have been considered Mars model microorganisms and the most primitive microorganisms on early Earth [40,41,42]. However, the sensitivity of halophilic methanogenic archaea to sole UVC radiation has not been examined thus far. Therefore, the study of the slightly halophilic methanogenic archaea not only provides the insight into the possibility of life on Mars, but also provides understanding for the evolution of life during early UV irradiated Earth. Most methanogenic chemolithoautrophic anaerobic archaea consume CO_2_ for their carbon source and H_2_ for their energy source and produce CH_4_ as their end product of metabolism. The various possible sources of carbon and hydrogen for methanogens on Mars have been discussed previously [21].

In this research, we irradiated hydrated and desiccated cultures of slightly halophilic and non-halophilic methanogenic archaea with a wide range of UVC (254 nm) flux, simulating the Martian UVC flux conditions, to examine their survivability.

## 2. Materials and Methods

### 2.1. Preparation of Growth Media

Pure cultures of *Methanobacterium formicicum* OCM55 and *Methanococcus maripaludis* OCM 151 were obtained from the Oregon Collection of Methanogens, Portland State University, Portland. *M. maripaludis* is slightly halophilic by definition (requiring 1–5% NaCl; [43]). It was isolated from a salt-marsh sediment and requires salt in its growth medium [44]. MSH medium (see below) contains approximately 3.4% total salt (about 3% NaCl), compared to the MSF medium for *M. formicicum*, the non-halophile, which contains less than 0.3% total salt (0% NaCl). Formulas for the two media are as follows:

(i) MSF medium [45] for *M. formicicum* contains per liter: 4.0 g NaOH, 0.25 g Na_2_S·9H_2_O, 1.0 g NH_4_Cl, 0.4 g K_2_HPO_4_·3H_2_O, 1.0 g MgCl_2_·6H_2_O, 0.4 g CaCl_2_·2H_2_O, 1.0 mg resazurin, 5.0 mg Na_2_-EDTA·2H_2_O, 1.5 mg CoCl_2_·6H_2_O, 1.0 mg MnCl_2_·4H_2_O, 1.0 mg FeSO_4_·7H_2_O, 1.0 mg ZnCl_2_, 0.4 mg AlCl_3_·6H_2_O, 0.3 mg Na_2_WO_4_·2H_2_O, 0.2 mg CuCl_2_·2H_2_O, 0.2 mg NiSO_4_·6H_2_O, 0.1 mg H_2_SeO_3_, 0.1 mg H_3_BO_3_, 0.1 mg NaMoO_4_·2H_2_O, 2.0 g yeast extract, 2.0 g trypticase peptone, 0.5 g mercaptoethanesulfonic acid and 0.25 g sodium formate. The medium was saturated with CO_2_ gas for 30 min using a gassing manifold, resulting in a pH of 6.7, and providing a carbon source.

(ii) MSH medium [46] for *M. maripaludis*, is MSF medium without sodium formate, but with the addition of 29.5 g NaCl, 1.7 g MgCl_2_·6H_2_O and 0.5 g KCl added per liter.

One hundred milliliters of each medium were prepared in a flask inside a Coy Laboratories anaerobic chamber (Coy Laboratory Products Inc., Grass Lake Charter Township, MI, USA), which was filled with 90% CO_2_ and 10% H_2_. CO_2_ in the chamber ensures that the media are saturated with CO_2_, which allows for a final pH of 6.7 and provides a carbon source. Also, CO_2_ is the predominant gas in the Martian atmosphere, and our research involves simulations of Martian conditions. MSF and MSH media were then transferred into anaerobic culture tubes (10 mL in each tube) inside the chamber. These tubes were then sealed with butyl rubber stoppers, removed from the chamber, crimped with an aluminum cap, and autoclaved for sterilization [18].

### 2.2. Preparation of Stock Cultures of Methanobacterium formicicum and Methanococcus maripaludis

Stock cultures of methanogens were maintained by transferring into their respective fresh growth media every fifteen days. Actively growing methanogenic cells (approximately 0.5 optical density at 600 nm) were utilized for UV irradiation either in solution or desiccation experiments. A sterile sodium sulfide solution (2.5% wt/vol; 0.125 mL per 10 mL of media) was added to each of the MSF and MSH media tubes about an hour prior to inoculation with the methanogens [45] in order to eliminate any residual molecular oxygen. One half of a milliliter from the *M. formicicum* or *M. maripaludis* stock was inoculated into each type of medium. After inoculating methanogens into their respective media, the tubes were pressurized with 200 kPa of hydrogen gas and incubated at their optimal growth temperature, 37 °C. Methane concentration in the headspace gas of each sample was measured periodically using a gas chromatograph (Shimadzu 2014, Kyoto, Japan) with a flame ionization detector, helium as carrier gas, and a Supelco custom packed column (80/100 HAYESEP Q, 2M X 1/8IN MM SS).

### 2.3. Preparation of Liquid Methanogenic Cells in Cuvettes for UV Irradiation

A sterile syringe was used to transfer 1 mL of an actively growing cell suspension (approximately 0.5 OD at 600 nm) to a sterile disposable plastic vial inside the anaerobic chamber. From the vial, 200 uL of aliquot were transferred into a plastic cuvette (44 mm × 12 mm × 12 mm; VWR, Radnor, PA, USA).

### 2.4. Preparation of Desiccated Methanogenic Cells in Cuvettes for UV Irradiation

From the same plastic vial in the previous section, 200 uL of aliquot were transferred into plastic cuvettes. These cuvettes were placed into a Nalgene Desiccator (150 mm, VWR Scientific Products, Dallas, TX, USA) containing Drierite (anhydrous calcium sulfate, W. A. Hammond Drierite Company, Ltd., Xenia, OH, USA) for about 72 h. All work was done inside an anaerobic chamber.

### 2.5. Exposure of Hydrated and Desiccated Methanogenic Cells to UVC Irradiation under Anaerobic Conditions

A small UVC lamp (UVG-11|compact UV lamp|4 watt|254 nm| P/N 95-0016-14|0.16 Amps|115 V~60 Hz) was used to irradiate methanogenic cells inside the anaerobic chamber. The dimension of the compact lamp was 7.8 L × 2.8 W × 2.1 D in. (198 mm × 71 mm × 53 mm). The lamp was placed 3 inches above the sample in the cuvette. The intensity of UVC flux, measured using a UVC light meter (UVC Light Meter 850010, Sper Scientific, Scottsdale, AZ, USA), was in the range of 7–9.9 Wm^−2^ (25.2–35.64 KJ m^−2^ h^−1^). The cuvette was placed at the same spot where flux was measured in order to calculate accurate flux experienced by the methanogens. The cuvette containing either liquid or desiccated cells was placed upright below the UVC lamp for various times ranging from 1 to 24 h. Thus, cultures were directly exposed to the UV light coming into the cuvette through the open end. A cuvette was chosen because it had a flat bottom, allowing it to stand upright under the lamp. The chemical composition of the cuvette was unimportant because the UV light was coming in through the open end. Control cells were treated exactly the same as the UV-exposed cells, but received no UV exposure. There was no mixing of the liquid cultures during the exposure procedure so as to avoid disruption in the time of exposure. All experiments were performed at ambient temperature and pressure. After exposure for the desired time intervals, 1 mL of the respective sterile growth media was added to each cuvette followed by thorough mixing. These 1 mL aliquots were transferred into their respective growth media with the use of a sterile syringe. Inoculated methanogenic media tubes were removed from the anaerobic chamber, pressurized with 200 KPa of H_2_, and incubated at their optimum growth temperature, 37 °C. One milliliter of a headspace gas sample was removed periodically to measure methane concentration using gas chromatography. The entire set of experiments was performed twice.

All the experiments were performed in the anaerobic chamber for three reasons: (i) methanogens are anaerobes; (ii) to mimic the Martian surface environment in terms of oxygen concentration; (iii) atmospheric oxygen can interfere with the effect of UVC radiation on cells. To normalize the experimental protocol in each set of experiments, the same OD and same volume of exponential phase cells of each species in liquid culture were utilized. In the desiccation experiments, we used the same volume of the liquid cultures to prepare desiccated cells. 

## 3. Results

The slightly halophilic methanogen, *M. maripaludis,* while hydrated survived for about 24 h of UVC exposure, while desiccated cells endured for 16 h (Table 1). The non-halophilic methanogenic archaeon, *M. formicicum*, also survived UVC radiation for 24 h in the hydrated state, however, while desiccated, the survivability of *M*. *formicicum* was 12 h (Table 1). The survivability of the methanogens was determined by measuring methane concentrations in headspace gas samples using gas chromatography after re-inoculation of methanogens into their respective growth-supporting media following exposure to UVC radiation. Under ideal control conditions, these two methanogens typically reach methane concentrations between 25% and 40% of the headspace gas with time. In Table 1, highest methane concentrations are recorded. In all cases, there was no measurable methane in any tube when first re-inoculated. Gas chromatographic analysis of methane occurred weekly for six weeks following re-inoculation. In eight cases, only one tube out of two demonstrated any methane (e.g., *M. formicicum* in liquid medium after one hour of UVC exposure). Where the highest methane concentration was recorded as 0, both tubes demonstrated no methane production during the six-week period.

## 4. Discussion

We studied the effect of simulated Martian UVC radiation on the survivability of hydrated and desiccated cultures of two methanogens, *M. formicicum*, a non-halophile, and *M. maripaludis*, a slight halophile. Mars is an extremely dry planet and any putative microorganism on the Martian surface might have to withstand a period of desiccation. However, the possibility of intermittent briny water on the surface of Mars has also been described [47]. Therefore, life on Mars could be in an anhydrobiotic or a hydrated state. Anhydrobiosis is a condition in which an organism enters into a completely desiccated state with reduced or no metabolic activity. We have reported earlier that *M. maripaludis* survived desiccation for 60 days while *M. formicicum* survived for 120 days at Martian surface or near surface pressure conditions, 6 mbar [48].

In order to determine the survival capability of the hydrated and the desiccated methanogenic cells under simulated Martian UVC radiation, we exposed cells to the radiation for various time intervals from 1 to 24 h. Methane concentrations from headspace gas samples following UVC exposure were measured using gas chromatography. Growth of methanogens is typically measured by increases in methane concentration with time [49]. Both methanogens, *M. formicicum* and *M. maripaludis*, in liquid culture endured UVC radiation for 24 h. However, when desiccated, *M. formicicum* and *M. maripaludis* tolerated ultraviolet radiation for 12 and 16 h, respectively. In a few cases, only one out of two cultures demonstrated methane production (Table 1). This is not unusual. In other research with these same methanogens [50], we saw very different responses in replicates, including high methane in one tube, low methane in another, and no methane in a third tube under identical conditions. This variation in methane concentrations can also be seen in the tubes that have large standard deviations in Table 1 (e.g., *M. formicicum* in liquid after 16 h of exposure; 18.3 ± 15.3% methane; actual methane readings, 3.3% and 33.9%). With respect to lag time, most of the cultures had greater lag times following any exposure to UVC compared to the controls, but there was no correlation between length of lag time and length of exposure, as might be expected. Also, liquid cultures were not mixed during UV exposure, so there was most likely some settling of cells, especially as the exposure time increased. This may have resulted in less exposure to cells sitting in the bottom of the liquid. A sample size of 200 uL was chosen to minimize the depth of liquid in the cuvette (1.5 mm at the meniscus).

One possible reason for the survivability of methanogens for 24 h of UVC exposure in the liquid culture could be the presence of suspended solids or other components in the growth media that prevented the damaging effect of the radiation on the cells. Some elements or minerals such as iron, sulfur, solid NaCl, jarosite (iron sulfate), and gypsum (calcium sulfate) as well as the organic components of the media can act as shielding agents and protect cells from the damage caused by ultraviolet radiation [10,51,52,53]. Iron sulfate was present in both growth media. The total salt concentration in the growth medium of *M. maripaludis* is only about 3.4%. The presence of salt in the MSH medium may have imparted protection to *M. maripaludis* in both liquid medium and the desiccated state. The minerals such as salt, iron compounds, and jarosite are common on the surface on Mars and could serve as protective materials for any putative life form there as well. Also, the presence of any overlying material or regolith could attenuate the effect of radiation on organisms and could increase the potential habitability of life on Mars. Furthermore, the microbes in the upper layer of a microbial mat can also protect microbes beneath them [53]. 

In the research reported here, the slightly halophilic archaeon *M. maripaludis* appeared to be more tolerant to ultraviolet radiation than non-halophilic *M. formicicum.* The reasons for the relative tolerance of methanogens to the UVC radiation are not known. One possible explanation could be the difference in their genomic structure and utilization of DNA repair enzymes. Since enzymes require an aqueous environment for activity, one would expect DNA repair to occur only in the hydrated cells. 

We have not attempted to simulate actual Martian surface conditions such as low pressure, low temperature, atmospheric gas composition or the presence of minerals along with the UVC radiation. We considered only two of the stress conditions, UVC radiation and desiccation. The results presented here illustrate that slightly halophilic and non-halophilic methanogens, in their hydrated state, tolerated simulated Martian UVC radiation longer than in their desiccated state. Future work will examine possible adaptation to UVC radiation by methanogens.

## 5. Conclusions

The research reported here illustrates that hydrated and desiccated cultures of *M. maripaludis* and *M. formicicum* could tolerate simulated Martian UVC flux for limited lengths of time. We irradiated the liquid and desiccated cultures of slightly halophilic *M. maripaludis* and non-halophilic *M. formicicum* to UVC (254 nm) flux for several time intervals. In the hydrated state, both strains of methanogens survived for 24 h of UVC exposure while in the desiccated state, *M. maripaludis* endured for about 16 h and *M. formicicum* survived for 12 h. The reasons for the survivability of these methanogens over various time intervals in the hydrated and desiccated conditions are not known. However, some components in the growth media and/or the activity of DNA repair enzymes could have protected cells from the damage normally caused by ultraviolet radiation. Therefore, limited exposure (12–24 h) to UVC radiation on the surface of Mars is not necessarily a limiting factor for the survivability of organisms such as *M. maripaludis* and *M. formicicum.*

## Figures and Tables

**Table 1:** Highest methane concentrations (%) of headspace gas produced by *Methanobacterium formicicum* and *Methanococcus maripaludis* following exposure to various time intervals of UVC radiation in liquid media or desiccated.

	*Methanobacterium formicicum*		*Methanococcus maripaludis*	
Exposure Time (h)	Liquid	Desiccated	Liquid	Desiccated
0 (Control)	21.7 ± 10.1	31.2 ± 1.7	28.1 ± 0.1	27.3 ± 0.4
1	22.1 ^a^	31.6 ± 1.4	14.6 ± 12.3	26.3 ± 0.4
2	31.3 ± 1.7	29.6 ± 0.2	24.0 ^a^	27.4 ± 0.7
4	28.8 ± 7.4	36.2 ± 0.4	26.3 ^a^	24.3 ± 0.7
8	27.6 ± 1.9	22.4 ^a^	14.7 ± 13.5	24.8 ^a^
12	30.2 ± 0	29.8 ± 1.1	26.2 ^a^	28.2 ^a^
16	18.6 ± 15.3	0	28.2 ± 2.0	27.2 ± 1.2
24	37.2 ± 4.0	0	23.6 ^a^	0

^a^ Only one culture out of two demonstrated methane production following exposure to UVC, thus no standard deviation.
